# Acute beetroot juice supplementation enhances short duration high-intensity exercise performance and influences muscle oxygenation in football players

**DOI:** 10.1038/s41598-026-37514-x

**Published:** 2026-01-31

**Authors:** Melike Nur Eroglu, Beril Kose, İpek Eroglu Kolayis, Bahtiyar Haberal

**Affiliations:** 1 Coaching Education Department, Faculty of Sports Science, Sakarya University of Applied Sciences, Sakarya, Turkey; 2 Nutrition and Dietetics Department, Faculty of Health Sciences, Baskent University, Ankara, Turkey; 3 Faculty of Medicine, Baskent University, Ankara, Turkey

**Keywords:** Beetroot juice, Football, High intensity exercise, Muscle oxygenation, Nitrate, Health care, Medical research, Physiology

## Abstract

Beetroot juice (BJ), a nitrate-rich supplement, may enhance exercise performance, but its acute effects on anaerobic power and muscle oxygenation in football players are unclear. This study investigated these effects during a Wingate test in trained male footballers. In a randomized, crossover, double-blind, placebo-controlled design, 16 male football players (age: 18.2 ± 0.4 years, weight: 69.6 ± 6.5 kg, height: 177.5 ± 5.5 cm) consumed 140 mL of BJ (12.8 mmol nitrate) or a placebo (blackcurrant juice (< 0.1 mmol nitrate)). After 2.5 h, a 30-s Wingate test was performed. Muscle oxygenation, heart rate, blood pressure, and blood lactate were assessed. BJ supplementation increased peak power (placebo: 720.33 ± 107.78 W; BJ: 803.50 ± 127.23 W, *p* = 0.002) and mean power (placebo: 541.23 ± 62.05 W; BJ: 581.42 ± 80.09 W, *p* = 0.001), and reduced the time to peak power (placebo: 7.32 ± 0.84 s; BJ: 8.10 ± 0.94 s, *p* = 0.032) compared to placebo. Muscle oxygenation did not differ during exercise (*p* > 0.05); however, post-exercise muscle oxygen saturation was higher (∼10%, *p* = 0.017) and deoxygenated haemoglobin was lower (∼13%, *p* = 0.019) with BJ. Post-exercise blood lactate levels were higher in BJ compared to placebo (*p* < 0.05). Blood pressure at all time points and heart rate during exercise did not differ between conditions (*p* > 0.05). Acute BJ supplementation increases anaerobic power output and post-exercise muscle oxygenation in football players, without affecting heart rate or blood pressure.

*Trial registration* The randomized controlled trial was registered on 03/07/2025 at ClinicalTrials.gov, under the registration number NCT07048912.

## Introduction

Nitric oxide is a key signaling molecule that regulates multiple physiological processes, such as mitochondrial respiration and biogenesis, vascular tone, glucose uptake in skeletal muscle, angiogenesis, and calcium handling within the sarcoplasmic reticulum^1^. Increased nitric oxide availability enhances skeletal muscle blood flow through vasodilation, improves oxygen delivery to active tissues, and reduces the oxygen cost of submaximal exercise by improving mitochondrial efficiency^2^. As these processes support various physical functions, maximizing nitric oxide production and availability may support exercise performance. Notably, dietary intake of nitrate-rich foods serves as a substrate for the nitrate–nitrite–nitric oxide pathway, through which nitrate is sequentially reduced to nitrite and subsequently to nitric oxide^3^.

The ergogenic potential of dietary nitrate supplementation was first highlighted by Larsen et al., who demonstrated a reduction in oxygen cost during submaximal exercise following sodium nitrate intake^4^. Since then, numerous studies have explored its effects across different exercise modalities, showing benefits in endurance performance and, in high-intensity, anaerobic activities^5–8^. Notably, these benefits are believed to be more pronounced in Type II muscle fibers, which are predominantly recruited during high-intensity, short-duration exercise such as sprint^9^. In these fibers, nitric oxide has been shown to enhance calcium release from the sarcoplasmic reticulum and reduce phosphocreatine degradation, thereby improving energy efficiency and delaying fatigue^1,10^.

Football is a dynamic sport that requires high-intensity and repetitive sprints, sudden accelerations, decelerations, changes of direction, and explosive power^11,12^. Throughout a match, players frequently exert maximum or submaximal effort, necessitating the efficient utilization of anaerobic energy systems. Anaerobic capacity is a critical determinant for football players in sustaining repeated sprints and maintaining superior performance during high-intensity phases^13^.

In this context, the Wingate test is a widely used method for assessing anaerobic power and capacity in sports where anaerobic energy production is crucial, such as football^14^. The 30-s maximal cycling effort enables the assessment of anaerobic power, fatigue index, and peak power. Although the test does not replicate football-specific movements, it is a validated measure of anaerobic capacity^15,16^, which underpins repeated sprint ability and recovery. Therefore, investigating football players with this protocol can provide meaningful insight into the ergogenic potential of beetroot juice (BJ) in sports where anaerobic performance is decisive.

During high-intensity exercise, the oxygen consumption by muscles surpasses the oxygen supply, leading to a decrease in muscle oxygenation levels^17,18^. Shortly after the cessation of exercise, the oxygen delivery to the muscles exceeds consumption, resulting in the restoration of muscle oxygenation^19^. However, the time required for reoxygenation post-exercise depends on the balance between oxygen delivery and muscular oxygen demand. Because nitrate supplementation has been suggested to facilitate oxygen transport and phosphocreatine resynthesis, its role in recovery after maximal efforts is of particular interest for intermittent team sports such as football^20^.

Several studies have demonstrated positive effects of acute nitrate supplementation on high-intensity exercise performance^21–24^. For instance, Cuenca et al. and Dominguez et al. reported improvements in peak power and time-to-exhaustion following BJ intake^21,22^. In contrast, other studies found no significant benefits during sprint or repeated sprint tasks^23,25–27^. These discrepancies suggest that the ergogenic potential of nitrate may depend on the type and intensity of exercise, the athlete’s fitness level, and supplementation strategy.

To better understand these inconsistencies, it is essential to examine not only performance outcomes but also the underlying physiological responses that may explain them, particularly muscle oxygenation dynamics during and after high-intensity exercise. However, few studies have specifically examined trained football players using both performance outcomes and real-time muscle oxygenation measures. This gap highlights the need for research integrating anaerobic performance tests with near-infrared spectroscopy (NIRS) to clarify the acute effects of BJ supplementation in this population.

Muscle oxygenation, a key determinant of anaerobic recovery and fatigue resistance, can be measured in real time using NIRS, a non-invasive technique that tracks changes in oxygenated and deoxygenated hemoglobin and myoglobin^28^. This method enables continuous monitoring of muscle oxygen saturation (SmO₂) during both exercise and recovery, providing valuable insight into the physiological mechanisms influenced by nutritional interventions such as nitrate supplementation.

Despite growing interest in BJ supplementation, its acute effects on muscle oxygenation during and after anaerobic protocols such as the Wingate test remain underexplored, particularly in trained football players. Given the inconsistent findings in the literature, examining both performance outcomes and real-time muscle oxygenation responses may provide novel insights into the physiological mechanisms underlying the ergogenic potential of BJ. Therefore, the aim of the present study was to investigate the effect of acute BJ supplementation on vastus lateralis SmO₂ during and after a 30-second Wingate anaerobic test in football players. It was hypothesized that acute BJ supplementation would enhance peak and mean power outputs and enhance muscle reoxygenation post-exercise, thereby serving as a potential ergogenic aid for short-duration, high-intensity efforts.

## Methods

### Participants

Sixteen male football players (age: 18.2 ± 0.4 years, weight: 69.6 ± 6.5 kg, height: 177.5 ± 5.5 cm, body fat: 6.4 ± 1.6%) participated in the study. All participants were members of the U-19 team of the Sakaryaspor Football Club. The participants were Tier 3 (Highly Trained/National Level) athletes, competing in national or state-level leagues^29^. Recruitment and study took place in July 2024. The inclusion criteria were: healthy males over 18 years old, performing resistance exercise at least three times per week, and having at least 5 years of football training experience. The exclusion criteria were individuals with contraindications to Wingate exercise, cardiometabolic disease, on ergogenic supplementation, and smokers. The participants took part in this study while they were in the season preparation camp and their training programmes were standardised as 4 days a week (12 h/week) during the study. All participants were informed of the potential risks and benefits associated with the study, and written informed consent was obtained. This study was reviewed and approved by the Baskent University Clinical Research Ethics Committee (ETH23/228) and conducted following the Declaration of Helsinki (2013).

### Randomization and sample size

An external researcher randomized the drinks of all participants to eliminate any order effects (Research Randomizer, https://www.randomizer.org*).* The sample size was calculated using the G*Power 3.1 software. The effect size (Cohen’s d = 1.35) was based on data from a previous study with a similar design^21^. According to the power analysis conducted with a significance level of α = 0.05 and statistical power of (1-β) = 0.80, a minimum total sample size of 16 participants was required.

### Experimental design

**Fig. 1:**
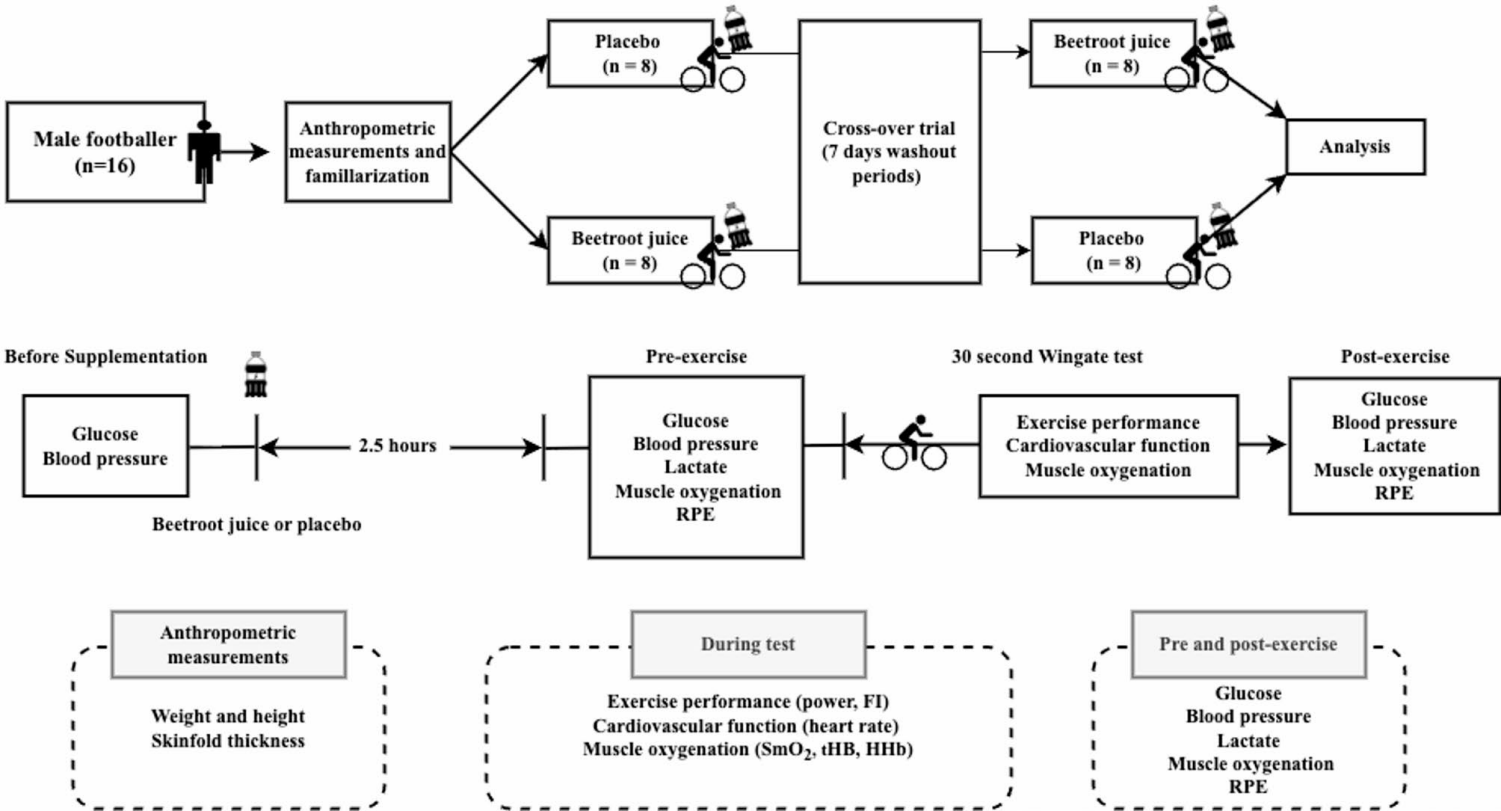
Schematic diagram of the experimental protocol FI, fatigue index; HHb, deoxygenated haemoglobin; RPE, rate of perceived exertion; SmO_2_, muscle oxygen saturation; tHb, total haemoglobin.

A randomized, double-blind, placebo-controlled and crossover experimental design was used in this study. Participants visited the laboratory 3 times. At the first visit, general information was obtained, and the Wingate familiarisation test was performed. Then, the participants were randomly divided into two groups. On each of the test days (second and third visits), the Wingate tests were performed 2.5 h after the nitrate-rich BJ or placebo supplementation^22^. At each test, the participants performed a 30-s Wingate test, and measurements were taken for muscle oxygenation, blood pressure, heart rate, ratings of perceived exertion (RPE), blood lactate and blood glucose. The washout period between the two supplementation periods was seven days. All testing sessions were conducted at the same time of day (± 1 h) and scheduled at least 48 h after the participants’ last training session to ensure full recovery and to minimize residual fatigue. The study procedure is illustrated in Fig. 1.

### Nutritional intervention and dietary control

Participants were asked to maintain their normal dietary and exercise behaviour throughout the study. However, participants were instructed to record their diet during the 48 h preceding the first Wingate test and to repeat this prior to the second Wingate test. To monitor compliance and minimize dietary variations, participants maintained a 48-hour food diary before each test session, which was reviewed by a dietitian. At the end of the study, food consumption records were analysed using the BEBIS software program (Ebispro for Windows, Stuttgart, Germany; Turkish Version BeBiS 8.2).

For 24 h before each test, participants were required to avoid high-intensity exercise, alcohol, caffeine, dietary supplements, nitrate rich foods (beetroot, celery, arugula, lettuce, spinach, turnip, chicory, Swiss chard, parsley, and cabbage) and anti-inflammatory medications. To ensure adherence, participants received a list of restricted vegetables prior to the study. Also, to prevent any attenuation in the reduction of nitrate to nitrite by commensal bacteria in the oral cavity, participants refrained from using antibacterial mouthwash/toothpaste and chewing gum 24 h before the supplementation.

Each participant consumed either 140 mL of the BJ (140 mL, 12.8 mmol/800 mg nitrate; Beet-It-Pro Elite Shot; James White Drinks Ltd., Ipswich, UK) or an equivalent volume of a placebo drink (low-calorie blackcurrant juice with negligible nitrate content (< 0.1 mmol), Pfanner, Austria)^30^. To maintain blinding, all study documents informed participants that the research aimed to evaluate the effects of natural, nutritious beverages on exercise performance. The specific details of the experimental design and the nutritional composition of the test and placebo drinks were disclosed only after study completion. The protocol was designed based on previous studies^3,7,8,31,32^.

After supplementation, participants were allowed to drink only water. The amount of water consumed before and during the first trial was replicated in the second trial to maintain consistency. They remained seated in a resting area under researcher supervision and were not permitted to engage in any physical activity or consume anything.

### Blinding procedure

To maintain the integrity of the study, a double-blind design was implemented, ensuring that participants and investigators were blinded to the supplement group allocations. Both the BJ and placebo supplements were placed in identical, dark, opaque bottles with matching color and design, labeled with randomly generated codes. An independent researcher, not involved in the data collection or analysis, handled the preparation and coding of the supplements. Investigators responsible for administering the supplements received only the coded containers and had no access to information regarding the contents of each container. This approach ensured that the investigators remained blinded throughout the intervention period, minimizing potential bias while administering supplements and assessing outcomes. This blinding procedure was strictly maintained until all data were collected and analyzed, at which point the group codes were revealed solely for statistical analysis purposes.

### Exercise protocol

Wingate protocol was used to determine anaerobic performance and fatigue. Test-retest reliability has consistently shown intraclass correlation coefficients ranging from 0.89 to 0.98, indicating excellent reproducibility across different populations and testing conditions^15^. For the test, a Monark cycle ergometer (Ergomedic 828E, Vansbro, Sweden) was used.

The Wingate test was started with the participant stopped^33,34^. Before the test, the following instructions were given by the investigators: (i) in the first seconds of the test, they should pedal from 0 rpm to the greatest pedalling velocity possible (rpm) in the shortest time possible; and (ii) maintain this high-power level during the longest time possible until the test end. During the familiarization session, participants were given detailed instructions on the correct positioning of the seat and handlebars to ensure optimal biomechanics for each individual. The seat height was adjusted to achieve a knee angle of approximately 170–175° for each participant, ensuring efficient pedaling mechanics. These personalized settings were maintained throughout all subsequent testing sessions to ensure consistency.

To further stabilize the participants’ legs, toe grips were used to keep their feet safe and stable, ensuring they remained in contact with the pedals at all times. Participants first performed a 5-min warm-up consisting of light cycling with the workload and cadence set by the participant followed by 1 min of rest. After this rest period, participants executed a specific warm up of 3 min of pedalling at a rate of 60 rpm with a workload of 2 kgf and a sprint at maximum intensity in the last 5 s of each minute. After 3 min of rest, the Wingate test was started.

The test consisted of 30 s of cycling at maximum effort with a load (kgf) corresponding to 7.5% of the participant’s body weight^15^. Participants were instructed to pedal as fast as possible to reach the maximum rpm in the shortest time possible and to try to maintain this pedalling speed until the end of the test.

Power output (W) was monitored second-by-second in all sprints (Monark Anaerobic Test Software 2.0, Sweden). Peak power and mean power were calculated by this software. The fatigue index (FI) was calculated using the equation: FI = (Wpeak − Wmin)/Wpeak × 100)^15^.

### Measurements

#### Muscle oxygenation variables

Muscle oxygenation was assessed using the NIRS technique with the Moxy Monitor (Fortiori Design LLC, Spicer, MN, USA), a portable and wireless device. The validity and reliability of the Moxy Monitor have been previously established. Crum et al. (2017) demonstrated that the device provides accurate and reproducible measurements of muscle oxygenation during incremental cycling exercise, showing strong to very large correlations for SmO₂ between repeated trials (*r* = 0.84–0.99, ICC = 0.77–0.99, *p* ≤ 0.01), thereby confirming its validity and suitability for exercise physiology research^35^. The Moxy Monitor utilizes four wavelengths of near-infrared light (680, 720, 760, and 800 nm) with a source-detector separation of 12.5 and 25.0 mm^36^. It measures three key parameters: muscle oxygen saturation (SmO₂), deoxygenated haemoglobin (HHb), and total haemoglobin (THb) during exercise.

Based on the different absorption spectra of oxygenated and deoxygenated haemoglobin + myoglobin in the near-infrared range, it provides SmO_2_ estimates as follows: SmO_2_ = 100 × [O_2_Hb]/[THb] ((oxygenated [haemoglobin + myoglobin]/total [haemoglobin + myoglobin]) × 100). This parameter is thought to be relatively insensitive to changes in blood volume and thus represents the balance between O_2_ delivery and utilisation at the measured site. HHb content, a parameter related to muscle O_2_ extraction, was calculated using the following equation: $[HHb]=[THb]-((SmO_2 \times [THb])/100)$ ^37^.

The vastus lateralis was chosen because of its major contribution to power generation during pedalling^35^. Moxy Monitor probes were placed on the right Vastus Lateralis muscle at two-thirds between the anterior superior iliac and the lateral side of the patella^38^. Each probe included the integrated light shield provided as part of the Moxy Monitor system, which minimizes interference from ambient light during measurement. Sensors were secured in place using medical adhesive tape.

The Moxy monitor provided wireless data at each interval throughout the test and these data were stored in the Moxy Monitor program for analysis. Baseline and post-exercise muscle oxygenation values were defined as the mean values of the athletes 2 min before warm-up and 2 min after the exercise.

#### Blood lactate variables

Blood lactate was measured at three time points: pre-exercise (2 min before warm-up), immediately post-exercise, and at the 3rd minute post-exercise. Capillary blood samples (5 µL) were obtained from the right index finger and analysed using a portable lactate analyser (LS, SensLab GmbH, Germany). The portable lactate analyzer used in this study has been previously validated as accurate and reliable for both laboratory and field testing (*r* = 0.97–0.99)^39^.

#### Blood glucose variables

Blood glucose was measured at three time points: immediately prior to supplementation, pre-exercise (2 min before warm-up), and immediately post-exercise. Measurements were performed using a portable glucose analyser (Plusmed Accuro, Taiwan), following the same sampling procedure used for lactate analysis.

#### Blood pressure variables

Blood pressure was measured at three time points: following a 10-minute rest upon arrival at the laboratory, prior to supplement consumption, pre-exercise, and at the 5th minute post-exercise. At each time point, three consecutive readings were taken, and the average of these readings was calculated and recorded for analysis.All measurements were conducted using an automated blood pressure monitor (Omron M2, Omron Healthcare Co., Kyoto, Japan). This device has been validated according to the European Society of Hypertension International Protocol, demonstrating excellent agreement with mercury sphygmomanometer measurements (mean difference = 2.7 ± 5.0 mmHg for systolic and − 1.4 ± 3.2 mmHg for diastolic blood pressure)^40^.

#### Heart rate variables

Heart rate was measured during exercise using the heart rate monitor (PolarV800, Kempele, Finland). Data were recorded via Bluetooth using the Elite HRV application (Heart Rate Variability, NC, USA), which served as the data receiver to capture the average heart rate^41^.

#### Rate of perceived exertion variables

The RPE of the participants were assessed using the 6–20 Borg Scale following the exercise protocol. Participants were instructed to report their perceived fatigue levels on this scale, which ranges from 6, indicating “no exertion,” to 20, representing “maximum effort” ^42^.

### Statistical analysis

The Shapiro-Wilk test was initially performed to assess the normality of the data distribution. Paired t-test was used for single measured variables. To analyse variables measured before supplementation, pre-exercise, and post-exercise, a two-way repeated measures analysis of variance (ANOVA) was conducted to evaluate the effects of supplementation (placebo vs. BJ), time (pre-supplementation, pre-exercise, post-exercise), and their interaction (supplement × time).

Bonferroni post hoc analysis was performed when a significant F value (Greenhouse–Geisser adjustment for sphericity) was observed. Effect sizes for ANOVAs were calculated using partial eta squared (ηp²), with thresholds defined as small (0.01), medium (0.06), and large (0.14) effects. Effect sizes for t-tests were measured using Cohen’s dz, with values categorized as small (0.2), medium (0.5), and large (0.8) effects^43,44^.

Statistical significance was set at *p* ≤ 0.05, and all data were presented as mean ± standard deviation (SD), unless otherwise specified. Statistical analyses were performed using the SPSS software (version 27.0, IBM, USA), and figures were made with GraphPad Prism software (version 10.0, GraphPad Software, USA).

## Results

### Dietary control

Participants were also asked at the end of the study whether they could tell which trial they were on to check allocation concealment. 75% said they did not know, and 25% thought they consumed placebo. No one said they consumed BJ. Of the participants who expressed an opinion, 50% were incorrect in their selection of supplement. Hence, the study blinding was effective. Also, when the dietary intakes of the participants were analysed, no significant difference was observed between the placebo and BJ conditions in energy (placebo: 2528.95 ± 336.90 kcal; BJ: 2664.85 ± 339.60 kcal, t = − 0.00, *p* > 0.05, d = 0.00), carbohydrate (placebo: 324.18 ± 68.25 g; BJ: 329.61 ± 68.25 g, t = 0.41, *p* > 0.05, d = 0.10), protein (placebo: 110.27 ± 19.89 g; BJ: 104.23 ± 20.57 g, t = 0.83, *p* > 0.05, d = 0.21) and fat (placebo: 98.52 ± 25.81 g; BJ: 99.04 ± 23.65 g, t = − 0.05, *p* > 0.05, d=-0.01) intakes.

### Wingate test performance

**Fig. 2:**
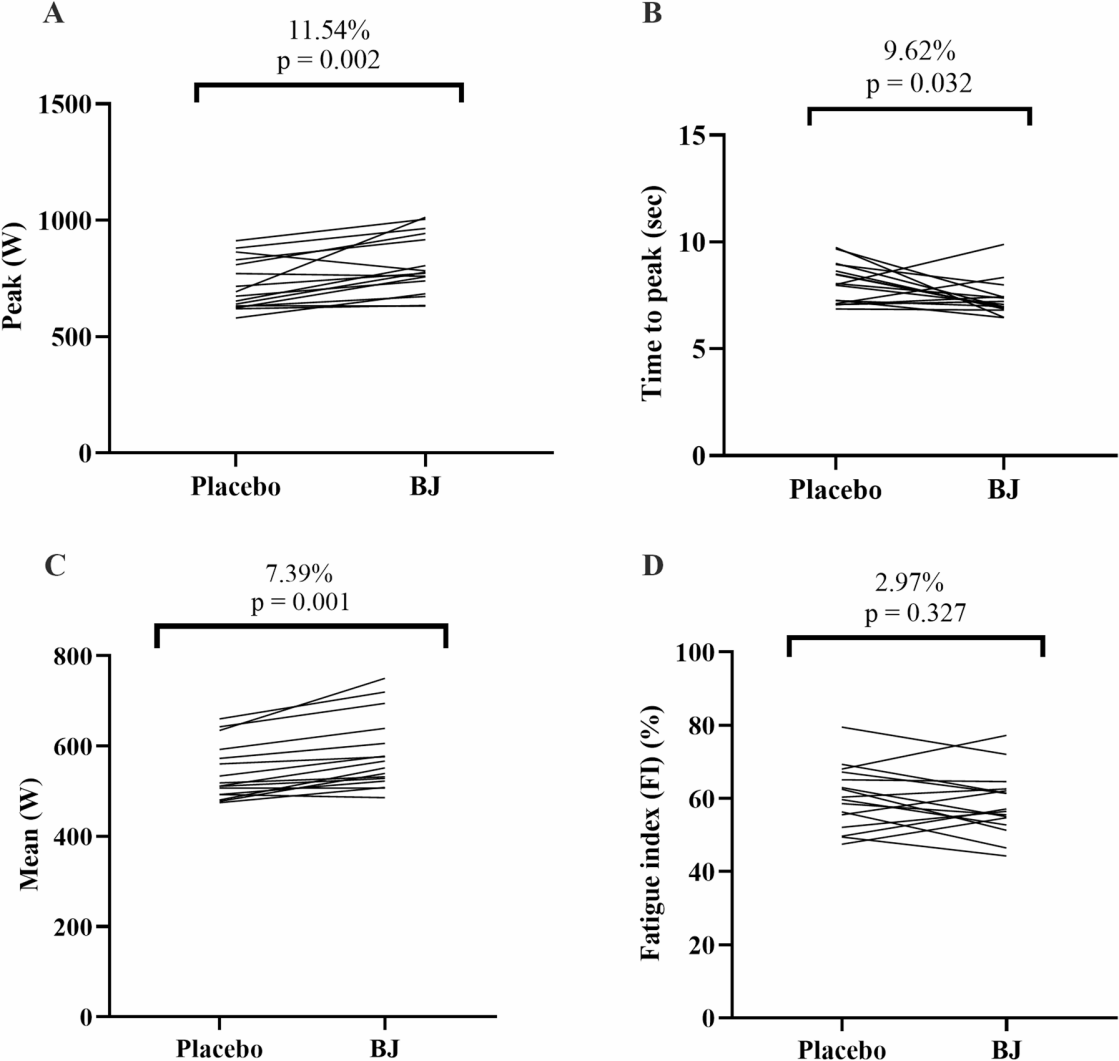
Effects of placebo or BJ intake on wingate performance peak power (**A**); time to peak (**B**); mean power (**C**); Fatigue index (FI) (**D**) BJ; beetroot juice.

Figure 2 illustrates the effects of placebo and BJ on the Wingate test. Mean power was significantly higher in the BJ condition (581.42 ± 80.09 W) compared to the placebo (541.23 ± 62.05 W) (t = 5.28, *p* < 0.05, d = 1.32). Significant differences were also observed between placebo and BJ for peak power (placebo: 720.33 ± 107.78 W; BJ: 803.50 ± 127.23 W; t = 3.72, *p* < 0.05, d = 0.93) and time to peak power (placebo: 8.10±0.94 s; BJ: 7.32±0.84 s; t = 2.36, *p* < 0.05, d = 0.59). However, no significant difference was found between placebo and BJ in FI (placebo: 58.44 ± 8.53%; BJ: 60.23 ± 8.54%; t = 1.03, *p* > 0.05, d = 0.34).

### RPE and heart rate

No difference was found between placebo and BJ in RPE (placebo: 16.5 ± 1.5; BJ: 15.4 ± 2.2, t = 1.41, *p* = 0.18, d = 0.88). No difference was observed between placebo and BJ in mean heart rate during exercise (placebo: 164.6 ± 5.4 bpm; BJ: 163.4 ± 7.6 bpm, t = 0.58, *p* = 0.57, d = 0.15).

### Muscle oxygenation

Muscle oxygenation data are presented in Table 1.No significant differences were observed between the placebo and BJ conditions in SmO₂ (*p* > 0.05, d = − 0.03), THb (*p* > 0.05, d = − 0.01), or HHb (*p* > 0.05, d = − 0.01) during exercise. While post-exercise THb was similar between the placebo and BJ (*p* > 0.05, d = 0.19), significant differences were found in SmO₂ (*p* < 0.05, d = − 0.68) and HHb (*p* < 0.05, d = 0.66).

**Table 1:**
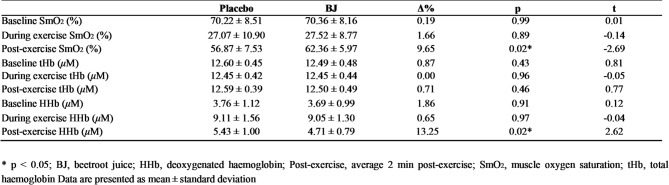
Effects of placebo or BJ intake on muscle oxygenation.

### Blood lactate

**Fig. 3:**
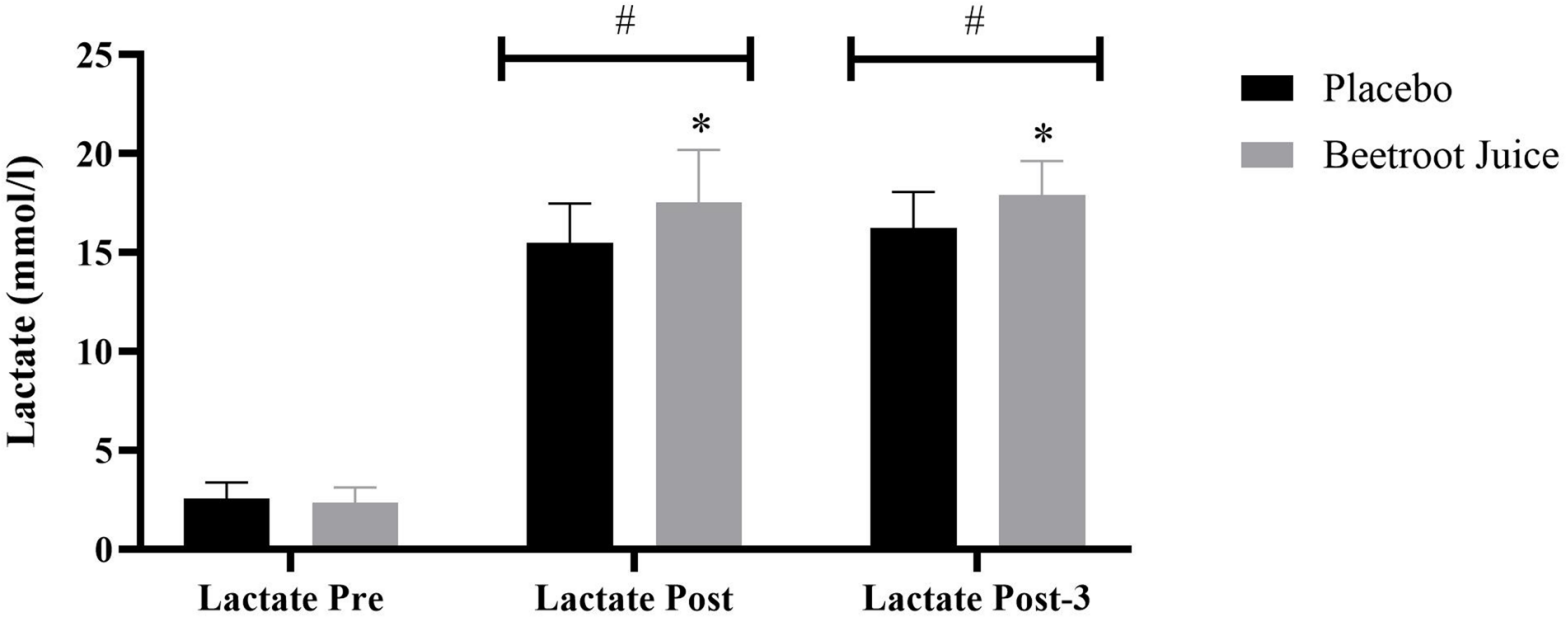
Blood lactate concentrations for placebo and BJ intakes pre, before exercise; post, immediately after exercise; Post-3, 3 min post-exercise. *Significantly different than placebo (*p* < 0.05). #Significantly different than pre-exercise (*p* < 0.05).

ANOVA revealed significant differences in blood lactate concentration (interaction effect: F = 7.95, *p* = 0.002, η² = 0.36; main effect of supplement: F = 6.12, *p* < 0.05, η² = 0.30) (Fig. 3). Blood lactate concentration was significantly higher after the exercise (main effect of time, *p* < 0.001). At pre-exercise, blood lactate concentrations were similar between placebo (2.61 ± 0.80 mmol/L) and BJ (2.39 ± 0.77 mmol/L) (*p* > 0.05). However, blood lactate concentrations were significantly higher in the BJ condition compared to placebo at post-exercise (placebo: 15.60 ± 2.01 mmol/L; BJ: 17.72 ± 2.63 mmol/L, *p* < 0.05) and 3 min post-exercise (placebo: 16.32 ± 1.87 mmol/L; BJ: 17.97 ± 1.76 mmol/L, *p* < 0.05).

#### Blood glucose and blood pressure

The effects of BJ and placebo on blood glucose, systolic blood pressure (SBP) and diastolic blood pressure (DBP) are shown in Table 2. There was no effect of supplementation on blood glucose, SBP and DBP (*p* > 0.05). However, 30-s Wingate test caused an increase in SBP (ANOVA time effect, *p* = 0.001) and a decrease in DBP (ANOVA time effect, *p* = 0.002).

**Table 2:**
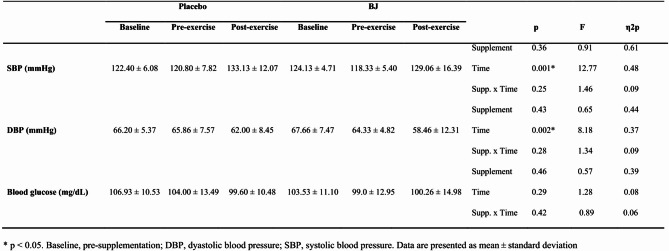
Effects of BJ or placebo intake on blood glucose and blood pressure.

## Discussion

This is the first study to investigate the effects of acute BJ on short-term high-intensity exercise performance in male football players. The primary findings are that acute BJ supplementation enhances 30-s Wingate test performance in trained football players by increasing mean and peak power while reducing the time to peak power. Additionally, BJ supplementation did not influence muscle oxygenation during exercise; however, it resulted in higher muscle oxygen saturation post-exercise. Furthermore, BJ supplementation led to elevated post-exercise blood lactate concentrations, whereas no significant effects were observed on blood pressure, heart rate, blood glucose levels, or RPE.

### Influence of BJ supplementation on 30-s wingate performance

This study confirms, for the first time in football players, that acute BJ supplementation improves Wingate performance. The primary finding shows that acute BJ supplementation enhanced 30-s Wingate test performance by increasing mean (~ 7%) and peak power (~ 11%), while reducing the time to peak power (~ 10%). Previous research has shown that BJ supplementation enhances blood flow, particularly in fast-twitch type II muscle fibers, thereby improving force production^1,45^. The potential mechanisms underlying this effect include the nitric oxide mediated enhancement of acetylcholine activity, which facilitates motor neuron depolarization, and the upregulation of calsequestrin expression, which promotes calcium release^1^. These mechanisms may increase calcium release from the sarcoplasmic reticulum, promoting stronger muscle contractions and greater power output^46^.

In addition to its role in enhancing power output in type II motor units, BJ supplementation has been reported to reduce ATP requirements during exercise^3,47^. This reduction has been observed as a decrease in phosphocreatine (PCr) depletion during both low- and high-intensity exercise^7^. A lower PCr cost during maximal efforts may delay fatigue^48^. Given the critical role of PCr in high-intensity exercise^49^, this delayed depletion may partly explain the 11% increase in peak power observed in this study.

Studies examining the effects of BJ supplementation on cycling sprint performance are limited and yield conflicting results. For instance, Cuenca et al. reported a 4% increase in mean and peak power and an 18% reduction in time to peak power in recreationally trained individuals following a 30-s Wingate test^21^. Similarly, Dominguez et al. found a 6.7% increase in mean power, a 5.4% increase in peak power, and an 8.4% reduction in time to peak power during the first 15 s of exercise^22^. Another study also reported a 4.4% increase in peak power and a reduction in time to peak power during the 30-s Wingate anaerobic test, although no differences were observed in mean power^23^. However, these studies differed in nitrate dose (ranging from 6.4 to 11.2 mmol), supplementation duration (acute vs. multi-day), and timing of intake (2–3 h prior to testing), which may partly explain the variability in results. The discrepancies across studies may also be attributed to differences in participant training status, as most previous studies recruited recreationally trained individuals, while the present study involved trained football players. Future research should aim to standardize these variables to better understand the ergogenic effects of BJ supplementation on high-intensity exercise performance.

### Influence of BJ supplementation on muscle oxygenation

This study is the first to examine the effects of acute BJ supplementation on muscle oxygenation during and post-Wingate test in football players. Our findings indicate that BJ supplementation did not significantly affect SmO₂ values during the exercise; however, post-exercise measurements revealed an increase in SmO₂ (~ 10%) and a decrease in HHb (~ 13%).

Previous research in both rats^9^ and humans^50^ has demonstrated that dietary nitrate enhances muscle blood flow via local vasodilation, potentially improving oxygen delivery to muscle tissues. It is well established that changes in oxygen availability can influence intracellular metabolism, metabolite accumulation, and ultimately muscle contractile function during exercise^51^. The existing literature on BJ supplementation and muscle oxygenation presents conflicting results, which may be attributed to variations in exercise protocols. For instance, previous studies have reported increased muscle oxygenation during transitions from low to high-intensity cycling and during high-cadence pedaling^51,52^. Research conducted under hypoxic conditions has yielded mixed findings, with some studies reporting preserved muscle oxygenation following nitrate supplementation^53,54^, while other studies found no significant effect^55–57^.

Consistent with these heterogeneous findings, the absence of differences in SmO₂ during maximal exercise in our study likely reflects physiological limitations of oxygen delivery under extreme intramuscular pressure. During maximal short-duration efforts such as the 30-s Wingate test, the rapid rise in intramuscular pressure transiently restricts muscle blood flow, resulting in near-maximal deoxygenation that may mask potential nitrate-related differences in oxygenation during exercise^58^. Consequently, the vasodilatory effects of nitric oxide are probably overridden by contraction-induced mechanical occlusion. Once exercise ceases and perfusion is restored, however, nitrate-mediated vasodilation and oxygen delivery become more apparent, resulting in higher SmO₂ and lower HHb in the BJ condition. This pattern is consistent with previous findings indicating that nitrate supplementation increases muscle contractile efficiency by reducing the phosphocreatine cost per contraction rather than increasing oxygen supply during maximal exertion^7,59^.

Collectively, these mechanisms suggest that the observed post-exercise improvement in muscle oxygenation arises from both enhanced oxygen delivery and improved metabolic efficiency. Studies investigating oxygenation during the recovery phase remain limited, but similar trends have been reported. For instance, in Jujutsu athletes BJ had no effect on SmO₂ during exercise but improved post-exercise oxygenation^60^, and Volino-Souza et al. observed faster muscle reoxygenation during recovery^61^. Our findings extend this evidence, suggesting that BJ supplementation may facilitate post-exercise reoxygenation, possibly through enhanced vasodilation, improved oxygen transport, and a lower energetic cost of PCr resynthesis.

Enhanced post-exercise reoxygenation may have practical implications for football performance. Faster restoration of muscle oxygenation may facilitate phosphocreatine resynthesis and metabolite clearance between high-intensity efforts, thereby supporting recovery during repeated sprints^62,63^. Although this study employed a single Wingate test, the observed recovery benefits suggest that BJ supplementation might also be advantageous in intermittent sports where rapid between-sprint recovery is critical^64,65^.

Finally, our findings also indicate that the low variation observed in THb values during and after the Wingate test may limit its utility as a precise marker of muscle oxygenation. Crum et al. suggested that THb may not be a valid indicator of oxygen utilization and distribution^35^. Therefore, a more comprehensive evaluation of muscle oxygenation should integrate THb data with more sensitive parameters, such as SmO₂.

### Influence of BJ supplementation on blood lactate

In our study, post-exercise blood lactate levels were higher following acute BJ supplementation. This finding aligns with previous research suggesting that BJ enhances glycolytic energy metabolism during high-intensity exercise^22,66^. Blood lactate concentration is a key biomarker of glycolytic metabolism, particularly in exercises that recruit Type II motor units and rely on high glycolytic activity^67^.

The observed increase in blood lactate levels after intense effort may be due to enhanced blood flow to Type II motor units induced by BJ supplementation^9^. Supporting this, Wylie et al. reported that BJ supplementation improved performance while increasing blood lactate levels during a cycling protocol with 6-second sprints and 24-second rest intervals^68^. Similarly, Mosher et al. found that BJ supplementation enhanced power output across repeated sets of bench press exercises at 60% of one-repetition maximum^69^. This effect is attributed to improved Type II motor unit performance, as these units depend more on glycolytic metabolism, leading to greater post-exercise blood lactate accumulation^70^.

### Limitations

Our study has some limitations. The ergogenic effect of nitrate supplementation are primarily attributed to its capacity to elevate plasma nitrite levels, which are subsequently reduced to nitric oxide^71^. Although plasma nitrite was not assessed following ~ 12.8 mmol dose in this study, previous research has consistently shown that even single doses as low as 6.4 mmol significantly increase plasma nitrite and improve exercise performance^7,66,67^. Therefore, it is reasonable to assume that double-dose protocol employed here effectively increased nitric oxide availability, even in the absence of direct biochemical assessment. While minor synergistic effects from other bioactive compounds in BJ (e.g., betalains, polyphenols, and antioxidants) cannot be excluded, their contribution is likely secondary given their limited bioavailability^72^ and the strong consistency of our findings with the nitrate–NO literature. Further, NIRS was placed only on the vastus lateralis, limiting the assessment of muscle oxygenation. To obtain a more complete picture of muscle oxygenation, future studies should consider assessing additional muscle groups.

## Conclusions

In conclusion, acute BJ supplementation significantly enhanced anaerobic performance in trained football players, as evidenced by increased peak and mean power and reduced time to peak power during the 30-s Wingate test. While no changes were observed in muscle oxygenation during exercise, a significant improvement in post-exercise muscle oxygen saturation was noted. These results suggest that BJ may facilitate recovery-related muscle oxygenation without altering exercise-phase oxygen dynamics. From a practical perspective, consuming BJ approximately 2.5 h before short-duration, high-intensity efforts may represent a simple and effective strategy to support performance and recovery in competitive settings.

## Data Availability

The data obtained from this study are included in the form of Tables and Figures. Further inquiries can be directed to the corresponding author.

## Abbreviations

ANOVA: Repeated-measures analysis of variance
BJ: Beetroot juice
DBP: Dyastolic blood pressure
FI: Fatigue index
HHb: Deoxygenated haemoglobin
NIRS: Near-infrared spectroscopy
PCr: Phosphocreatine
RPE: Rate of perceived exertion
SBP: Systolic blood pressure
SmO_2_: Muscle oxygen saturation
THb: Total haemoglobin
