# Correction: Acute beetroot juice supplementation enhances short duration high-intensity exercise performance and influences muscle oxygenation in football players

**DOI:** 10.1038/s41598-026-53213-z

**Published:** 2026-05-18

**Authors:** Melike Nur Eroglu, Beril Kose, İpek Eroglu Kolayis, Bahtiyar Haberal

**Affiliations:** 1 Coaching Education Department, Faculty of Sports Science, Sakarya University of Applied Sciences, Sakarya, Turkey; 2 Nutrition and Dietetics Department, Faculty of Health Sciences, Baskent University, Ankara, Turkey; 3 Faculty of Medicine, Baskent University, Ankara, Turkey

Correction to: *Scientific Reports* 10.1038/s41598-026-37514-x, published online 31 January 2026

The original version of this Article contained an error in the Results section.

As a result,

“Significant differences were also observed between placebo and BJ for peak power (placebo: 720.33 ± 107.78 W; BJ: 803.50 ± 127.23 W; t = 3.72, p < 0.05, d = 0.93) and time to peak power (placebo: 7.32 ± 0.84 s; BJ: 8.10 ± 0.94 s; t = 2.36, p < 0.05, d = 0.59).”

now reads:

“Significant differences were also observed between placebo and BJ for peak power (placebo: 720.33 ± 107.78 W; BJ: 803.50 ± 127.23 W; t = 3.72, p < 0.05, d = 0.93) and time to peak power (placebo: 8.10 ± 0.94 s; BJ: 7.32 ± 0.84 s; t = 2.36, p < 0.05, d = 0.59).”

The original Article has been corrected.

